# Pedicled Double‐Barrel Fibular Transplantation Versus Bone Transport in the Treatment of Upper Tibial Osteomyelitis with Bone Defects: A Retrospective Study

**DOI:** 10.1111/os.13466

**Published:** 2022-09-21

**Authors:** Qiang Huang, Yao Lu, Teng Ma, Qian Wang, ChaoFeng Wang, Zhong Li, Kun Zhang, Cheng Ren

**Affiliations:** ^1^ Department of Orthopedic Surgery, Hong Hui Hospital Xi'an Jiaotong University College of Medicine Xi'an China

**Keywords:** bone defects, bone transport, double‐barrel, fibular transplantation, tibial osteomyelitis

## Abstract

**Objective:**

This study aimed to compare the clinical effects of using pedicled double‐barrel fibular transplantation (PDBFT) and bone transport (BT) for the treatment of upper tibial osteomyelitis with bone defects.

**Methods:**

A total of 83 patients with upper tibial osteomyelitis and bone defects were selected and retrospectively studied in Xi'an Hong Hui Hospital from January 2009 to January 2019. There were 52 males and 31 females, aged 19–72 years. The tibial defect range was 5–12 cm. Patients were divided into two groups, including the PDBFT (40 cases) and the BT group (43 cases). All patients were classified according to Cierny–Mader classification, including 48 cases of type III and 35 cases of type IV. Operation time, blood loss and cure time were compared. Ennecking score was used to evaluate limb functions, including pain, activity function, self‐perception, brace use, walking ability, and gait change, while self‐rating anxiety scale (SAS) was used for postoperative mental and psychological status. In addition, complications were recorded. All patients were followed for at least 2 years. SPSS 23.0 software was used to process data.

**Results:**

There was no significant difference in demographic data between the two groups (*p* > 0.05). Operation time was 182.5 ± 22.7 min in PDBFT group *vs*, 124.2 ± 15.6 min in BT group, respectively (*p* < 0.05); intra‐operative blood loss was 286 ± 34 ml *vs* 45 ± 18 ml (*p* < 0.05); cure time was 7.3 ± 1.8 months *vs* 11.6 ± 3.7 months (*p* < 0.05); and Ennecking score was 87.3% and 76.0%, respectively (*p* < 0.05). So, the PDBFT group showed longer operation time, more blood loss, shorter cure time, and better Ennecking score than the BT group. Importantly, limb functions of the PDBFT group were better than that of the BT group. Moreover, the PDBFT group presented better postoperative mental status and fewer complications than that in BT group (*p* < 0.05).

**Conclusions:**

Patients were successfully cured by both the PDBFT and BT techniques. Compared with the BT group, the PDBFT group brought better clinical effects and fewer complications which could be the first operative choice for the treatment of upper tibial osteomyelitis with bone defects.

## Graphical abstract

Patients could be successfully cured by both pedicled double‐barrel fibular transplantation (PDBFT) and bone transport techniques. The PDBFT technique brought better clinical effects, which could be the first operative choice for the treatment of upper tibial osteomyelitis with bone defects.

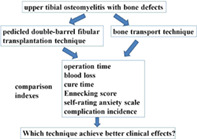

## Introduction

Soft tissues are weak at the tibia which easily leads to tibial osteomyelitis after a severe open fracture^1^. If the soft tissue protection is insufficient, it may also lead to infection and even osteomyelitis after an internal fixation^1^. While the treatment is not timely or infection cannot be effectively controlled, amputation may be unavoidable^2^, ^3^. Amputation brings lifelong disability to patients and greatly affects quality of life. In practice, such patients have a strong demand for limb salvage treatment. The treatment principle is staged surgeries, including controlling infection, soft tissue and bone reconstruction, and limb function recovery^4^. Although the treatment principle is clear, the treatment course is long. In this process, patients often undergo several operations, and have to tolerate great mental and psychological pressures.

Radical debridement is the most fundamental treatment, including removal of sinus tracts, all infected soft tissues and sequestrum^5^, ^6^. Bacterial culture is carried out during debridement. According to the culture results, large doses of antibiotics are applied for patients with tibial osteomyelitis. The antibiotic principle is early, sensitive, sufficient, and combined application. The debridement can stop until fresh bloods ooze from soft tissues and bone sections^7^, ^8^. Yet, different sizes of bone defects will appear after removing all sequestrum^9^.

Several techniques have been used in long segmental tibial defect reconstruction, such as Ilizarov bone transport, autologous bone transplantation, induced membrane technique, fibular transplantation, and so forth^10^, ^11^, ^12^, ^13^, ^14^, ^15^. Ilizarov technique is based on the principle of “distraction osteogenesis.” Through osteotomy and traction, new calluses gradually generate. In some cases, controlling infection and bone reconstruction can be carried out simultaneously. When the bone segment is lengthened, soft tissues, including skin, nerves, vessels, and tendons, grow and are lengthened in the meantime^16^. However, this technique brings lots of complications, such as pin‐tract infection, foot drop, axial deviation, skin incarceration, docking site nonunion, joint stiffness after operation, and so forth^10^, ^11^. The management for these complications significantly prolongs the treatment course, increases the number of operations and medical costs. In addition, wearing the Ilizarov fixator for a long time results in extreme discomfort to patients. These patients are difficult to quickly return to normal life and work^12^, ^13^. Autogenous bone grafting is usually applied to patients with a small amount of bone defects^17^. This limits its wide application. The induced membrane technique has been widely used for bone defect reconstruction in recent years. Compared with bone transport, patients with large segmental bone defects feel more comfortable by using the induced membrane technique^14^. Yet, due to the limited number of autogenous bones, and unavoidable bone resorption, sometimes induced membrane technique cannot fully solve the problem of long segmental bone defects^15^.

Fibular transplantation includes free and pedicled transplantation. It requires good microsurgical skills to perform a free fibular transplantation. A free fibular surgery usually takes a long time and has a lot of blood loss. Unlike free transplantation, the pedicled transplantation surgery is relatively simple with low risk. By breaking the fibula into a parallel double‐barrel shape, the grafted fibular strength will be significantly enhanced. This method has achieved good results in bone reconstruction of the upper tibial defects^18^, ^19^. Yet, it is still unclear whether the pedicled double‐barrel fibular transplantation is superior to bone transport.

The authors aimed to compare the clinical effects using PDBFT or BT technique for the treatment of upper tibial osteomyelitis with bone defects, and to clarify which method is better for patients with osteomyelitis and bone defects of the upper tibia.

## Methods

### Inclusion Criteria

(i) Patients over 18 years; (ii) Patients meeting the diagnostic criteria of upper tibial osteomyelitis; (iii) Patients treated by the PDBFT or BT technique and followed for at least 2 years; (iv) Patients with complete medical records; (v) Bone defects ranging from 5–12 cm.

### Exclusion Criteria

(i) Patients with major comorbidities and unable to tolerate anesthesia or surgery; (ii) Uncompleted clinical and follow‐up data; (iii) Patients got amputation treatment finally.

### General Data of Patients

A retrospective study was conducted. A total of 83 patients were selected in Xi'an Hong Hui Hospital from January 2009 to 2019. There were 52 males and 31 females, aged 19–72 years. After radical debridement, the upper tibial defects were 5–12 cm. A total of 40 patients were treated using the PDBFT technique and the other 43 cases using the BT technique. All patients were classified according to Cierny–Mader classification^20^. This study was approved by the ethics committee of Xi'an Hong Hui Hospital (No. 202207005). All patients or their family members signed the informed consent before operation.

### Preoperative Treatment

Computed tomography was performed for the injured limb. Blood samples were drawn to detect ESR, C‐reactive protein, and white blood cell count. All patients underwent radical debridement in the first stage. After removing all sequestrum, the residual tibial defects were filled with antibiotic‐loaded bone cement in PDBFT group. When the wounds healed and infection was effectively controlled, bone reconstruction was performed. In BT group, segmental resection method could be used during debridement.

### Operation Procedures of PDBFT Group

**Fig. 1:**
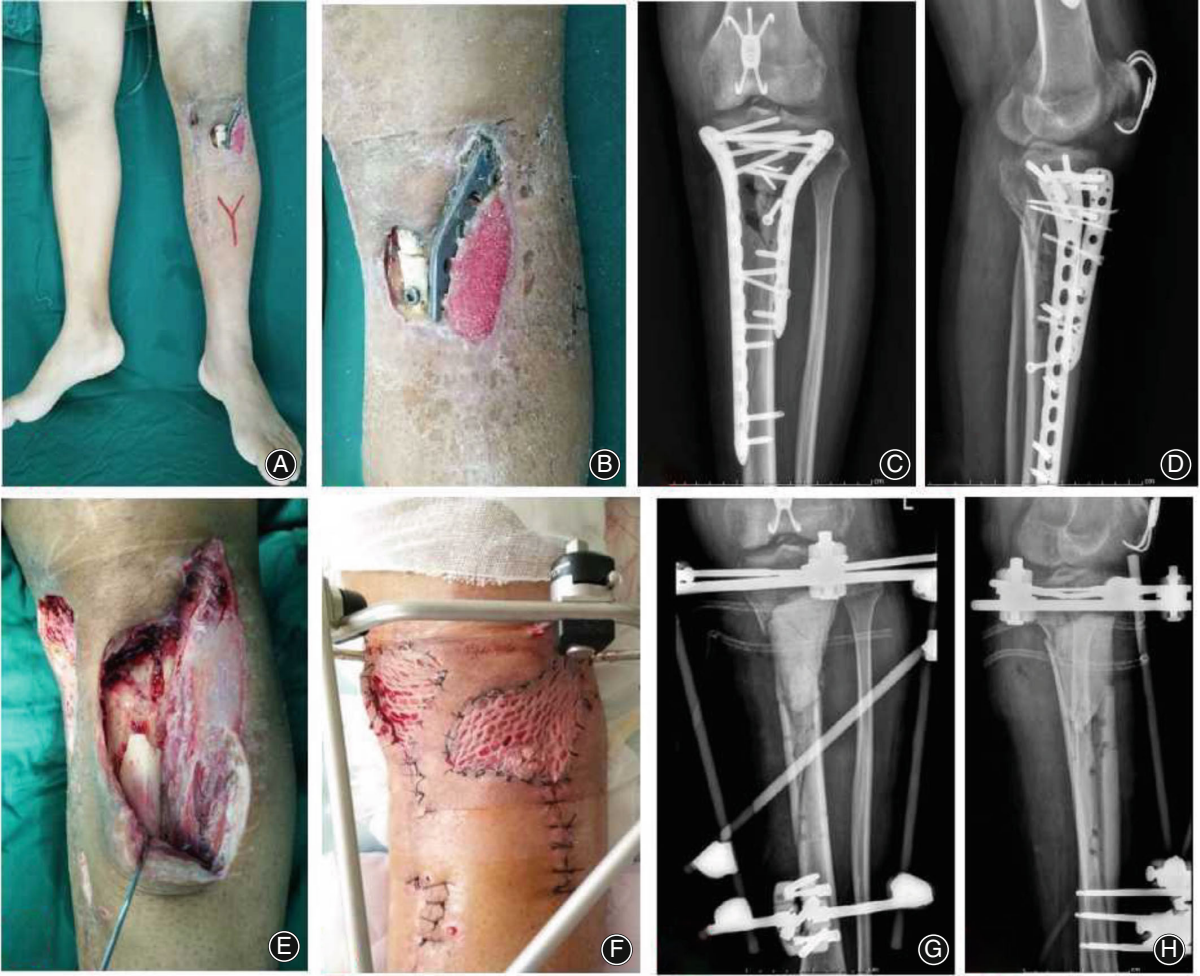
A 30‐year‐old male with infection and soft tissue defects after internal fixation of proximal tibial fracture. (A, B) Appearance before debridement; (C, D) X‐ray images of the proximal tibia before debridement; (E) After radical debridement, a large area of soft tissue and bone defects were left; (F) Appearance after transplantation of medial head of gastrocnemius muscle and skin grafting; (G, H) Postoperative X‐ray images showed that bone cement was filled into the proximal tibia

**Fig. 2:**
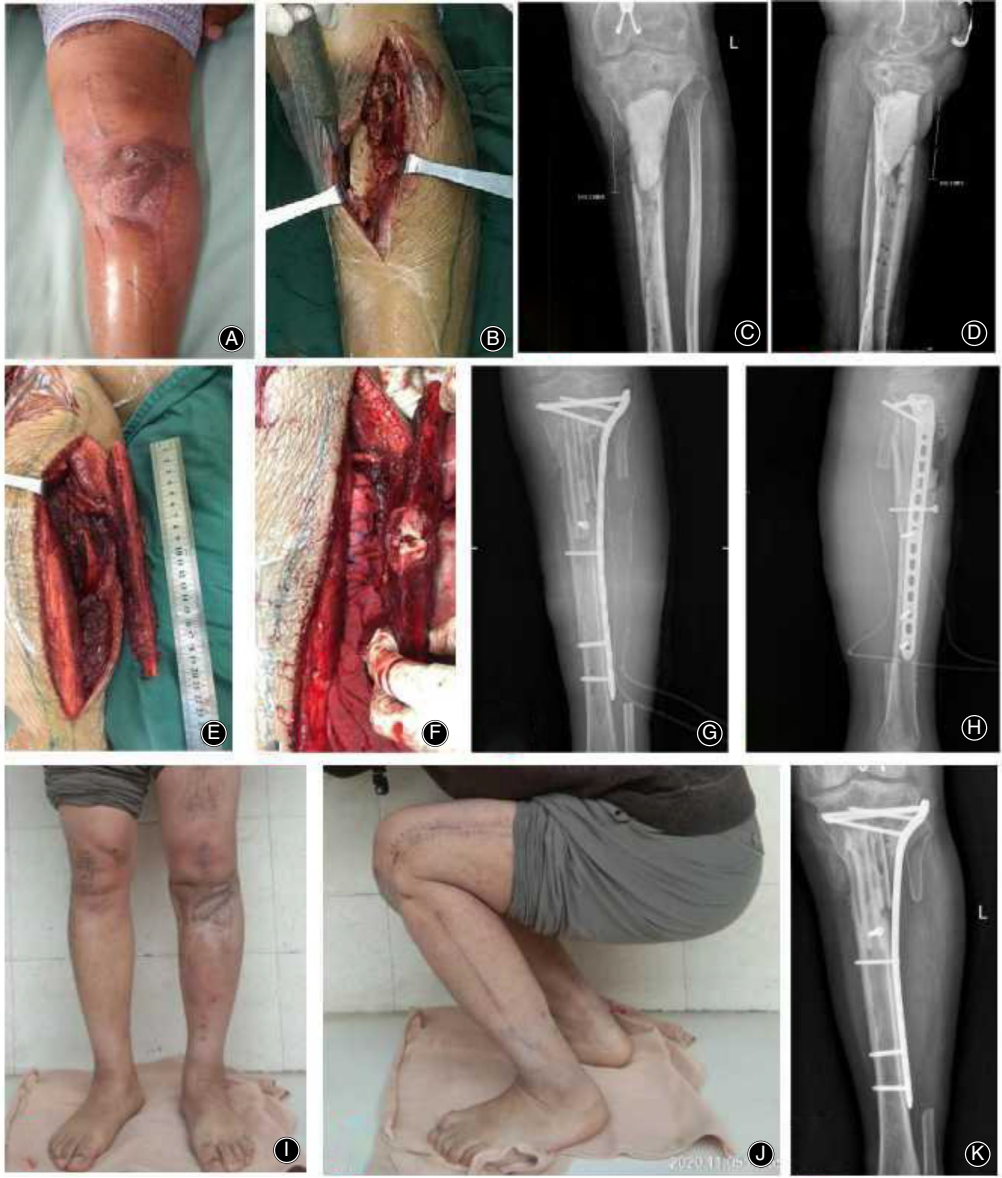
The 30‐year‐old patient was treated by the PDBFT technique. (A) Six weeks after debridement, the wounds healed well; (B) During second operation, bone cement was removed from the medial incision of the proximal tibia; (C, D) Preoperative X‐ray images showed that the tibial defects were about 10 cm; (E, F) During operation, the ipsilateral pedicled fibula was made into a double‐barrel shape for transplantation; (G, H) X‐ray images after the PDBFT was performed; (I, J) One year after operation, the appearance of standing and squatting position; (K) One year after operation, the grafted fibula healed well. PDBFT stands for the pedicled double‐barrel fibular transplantation.

**Fig. 3:**
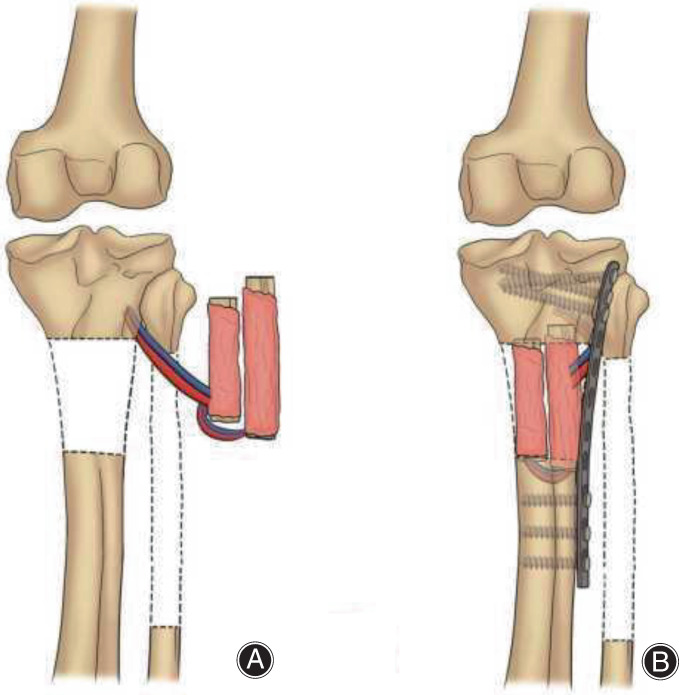
The key surgical procedures of the PDBFT technique. (A) The ipsilateral pedicled fibular flap was cut and folded into two segments for pedicled transplantation; (B) The pedicled double‐barrel fibular flap was transplanted into the defect site of the upper tibia, the distal and proximal tibia were bridged by a locking plate.

The patient was placed in supine position. Combined spinal epidural anesthesia or general anesthesia was taken. An anteromedial incision was taken to remove the bone cement. Then, another incision was made between the fibular head and lateral malleolus. It was entered from the space between the soleus muscle and fibular muscles. One centimeter of medial fibular cuff was retained. The peroneal artery and vein were included in the muscle cuff. The fibula was cut for required length. The peroneal artery and vein were cut off and ligated at distal part of the fibular flap. The proximal pedicle was properly freed for pedicled transfer. The fibular flap length was more than two times of the actual tibial defects, and 2–4 cm longer. According to the design, the fibula was cut into two unequal fragments. One was 2–3 cm longer than the other. Importantly, the continuity of medial muscle cuff was preserved. Distal part of the fibular flap was reversed for 180°. Then, a soft tissue tunnel was prepared and the tibial defect ends were slotted. The pedicled double‐barrel fibular flap was transferred to the defect site through the soft tissue tunnel. Attention was paid not to over twist the pedicle. The fibular flap was inserted vertically and stuck into the medullary cavity. It could be fixed to the tibia with screws. A locking plate was inserted to bridge tibial defects. The foot was kept in neutral position, and the flexor pollicis longus was fixed to the interosseous membrane. A typical case is shown in Figs 1 and 2. The key surgical procedures are shown in Fig. 3A,B.

### Operation Procedures of BT Group

The patient was placed in supine position and proper anesthesia was given. The injured leg was maintained in the center of the annular fixator with Kirschner wires, parallel to the knee and ankle articular surface, respectively. The transport ring was fixed at a suitable plane of distal tibia. Osteotomy was performed close to the distal tibia. Then, the alignment of bone defect ends was adjusted under a C‐arm image intensifier. One week after operation, the external fixator was adjusted for bone transport and the injured limb began to load partially on the ground. The initial lengthening speed was 1.0 mm per day for six times. When the bone defects were shortened within 4 cm, the speed was adjusted to 0.5–0.75 mm per day, which was completed in 3–4 times. After the two defect ends contacted, it was continued to press properly. This would accelerate the docking site healing. In the transport process, attention was paid to nerve and vessel status. If axial deviation occurred, it was adjusted in time. The fixator was removed when consolidation period finished and docking site firmly healed.

### Postoperative Management

Functional exercise was carried out postoperatively, including knee and ankle exercise. Symptomatic treatments were given, such as anti‐inflammatory, detumescence, and pain relief. Moreover, X‐ray films were performed to evaluate healing and bone transport process.

### Evaluation Indexes

Operation time, blood loss, and cure time were compared between the two groups. Cure time was defined as total time from admission to the time that bone defects were fully repaired. At 1 year follow‐up, limb functions were evaluated by Ennecking score^21^, including pain, activity function, self‐perception, brace use, walking ability, and gait change. Each item was scored 0–5 points, and the full score was 30 points. The cumulative score divided by 30 points was the percentage reaching normal limb functions. At 1 month after operation, SAS^22^ was used to evaluate the postoperative mental and psychological status, including no anxiety, mild anxiety, moderate anxiety, and so forth. In addition, complications were recorded, such as infection recurrence, nonunion, joint stiffness, and so forth.

### Statistical Analysis

SPSS 23.0 software (IBM Company) was used to process data. Measurement data were expressed as mean ± standard deviation. Unpaired *t* test was used for comparisons between the two groups. Count data were analyzed using *χ* ^2^ test. *p* < 0.05 was defined as statistically significant.

## Results

### Demographics

As shown in Table 1, the mean age was 35 ± 8 years in PDBFT group and 37 ± 7 years in BT group, respectively. There were 25 males and 15 females in PDBFT group, while 27 males and 16 females in BT group. The mean size of defects was 7.8 ± 2.3 cm in PDBFT group and 7.5 ± 2.5 cm in BT group. The mean number of previous operations was 3.2 ± 1.7 in PDBFT group and 3.4 ± 1.4 in BT group, respectively. According to Cierny–Mader classification, there were 23 type III and 17 type IV cases in PDBFT group, while there were 25 type III and 18 type IV cases in BT group. There were 57 defects caused by trauma and 26 by other reasons. In addition, single bacteria, including *Staphylococcus aureus*, *Pseudomonas aeruginosa* or *Escherichia coli*, was found in 51 cases while two or more kinds of bacteria were found in 32 cases. There was no significant difference in demographic information between the two groups (*p* < 0.05, Table 1). All patients were followed for at least 2 years.

**TABLE 1:** Demographics of patients

Variable	PDBFT group (*n* = 40)	BT group (*n* = 43)	χ^2^/t	*p* value
Age (year)	35 ± 8	37 ± 7	1.208	0.231
Sex			0.001	0.978
Male	25	27		
Female	15	16		
Size of defects (cm)	7.8 ± 2.3	7.5 ± 2.5	0.569	0.571
Previous operations	3.2 ± 1.7	3.4 ± 1.4	0.583	0.562
Cierny–Mader classification			0.003	0.953
Type III	23	25		
Type IV	17	18		
Etiology			0.050	0.824
Trauma	27	30		
Others	13	13		
Culture results			0.507	0.476
Single bacteria	23	28		
Two or more bacteria	17	15		

Abbreviations: BT, bone transport; PDBFT, pedicled double‐barrel fibular transplantation.

### Comparison of Operation Indexes

The mean operation time was 182.5 ± 22.7 min in PDBFT group and 124.2 ± 15.6 min in BT group, and the difference was significant (*p* < 0.05, Table 2). The mean intra‐operative blood loss was 286 ± 34 ml in PDBFT group and 45 ± 18 ml in BT group, with significant difference between the two groups (*p* < 0.05, Table 2). The mean cure time was 7.3 ± 1.8 months in PDBFT group and 11.6 ± 3.7 months in BT group, and the difference was significant (*p* < 0.05, Table 2).

**TABLE 2:** Operation indexes and functional scores

Variable	PDBFT group (*n* = 40)	BT group (*n* = 43)	χ^2^/t	*p* value
Operation time (min)	182.5 ± 22.7	124.2 ± 15.6	13.539	<0.05
Blood loss (ml)	286 ± 34	45 ± 18	39.926	<0.05
Cure time (month)	7.3 ± 1.8	11.6 ± 3.7	6.804	<0.05
Ennecking score			8.869	<0.05
26–30	20	9		
21–25	16	25		
16–20	4	7		
11–15	0	2		
SAS score			29.819	<0.05
No anxiety	29	7		
Mild anxiety	11	25		
Moderate anxiety	0	11		

Abbreviations: BT, bone transport; PDBFT, pedicled double‐barrel fibular transplantation; SAS, Self‐rating Anxiety Scale.

### Comparison of Ennecking Score

There was significant difference for Ennecking score between the two groups (*p* < 0.05, Table 2). Ennecking score in PDBFT group: 26–30 points, 20 cases; 21–25 points, 16 cases; and 16–20 points, four cases. The mean score was 26.2 points and 87.3% of normal functions was recovered in PDBFT group. Ennecking score in BT group: 26–30 points, nine cases; 21–25 points, 25 cases; 16–20 points, seven cases; and 10–15 points, two cases. The mean score was 22.8 points and 76.0% of normal functions was recovered in BT group. Ennecking score was significantly higher in PDBFT group than that in BT group (*p* < 0.05, Table 2).

### Comparison of Mental and Psychological Anxiety Index

There was significant difference in mental and psychological anxiety index between the two groups (*p* < 0.05, Table 2). SAS in PDBFT group: no anxiety, 29 cases; and mild anxiety 11 cases. SAS in BT group: no anxiety, seven cases; mild anxiety 25 cases; and moderate anxiety, 11 cases. The anxiety index was significantly lower in PDBFT group than that in BT group (*p* < 0.05, Table 2).

### Comparison of Complications

As shown in Table 3, in PDBFT group, there were two cases suffering from poor union after operation. The defects healed well after another autologous iliac grafting surgery. One patient had infection recurrence in PDBFT group. The patient underwent radical debridement again and then bone transport was performed. Five patients suffered from partial loss of joint movement. They were guided to take functional exercise or underwent surgical release. However, in BT group, there were 15 cases suffering from pin‐tract infection, four cases for foot drop, six cases for axial deviation, four cases for skin incarceration, eight cases for docking site nonunion, two cases for infection recurrence, and nine cases for knee joint stiffness. For patients with pin‐tract infection, routine dressing change was performed first. If it did not work, the infected Kirschner wires would be removed, and a new Kirschner wire was inserted. Patients with foot drop were given manual release or corrected by an external frame. Axial deviation was corrected by adjusting the annular frame. Patients with skin incarceration were treated by local skin and soft tissue release. In addition, patients with docking site nonunion were treated by removal of external frame, cancellous bone transplantation, and internal fixation with a plate. After 3 months, the tibial defects healed. Patients with infection recurrence were managed by radical debridement and sensitive antibiotics. Those with knee joint stiffness were treated by arthrolysis. The mean number of complications per patient was significantly lower in PDBFT group than that in BT group (*p* < 0.05, Table 3).

**TABLE 3:** Complications of the two groups

Variable	PDBFT group (*n* = 40)	BT group (*n* = 43)	t	*p* value
Pin‐tract infection	0 (0%)	15 (34.9%)	—	—
Foot drop	0 (%)	4 (9.3%)	—	—
Axial deviation	0 (0%)	6 (14.0%)	—	—
Skin incarceration	0 (0%)	4 (9.3%)	—	—
Docking site nonunion/poor union	2 (5.0%)	8 (18.6%)	—	—
Infection recurrence	1 (2.5%)	2 (4.7%)	—	—
Joint stiffness/partial loss of joint Movement	5 (12.5%)	9 (20.9%)	—	—
Number of complications per patient	0.20 ± 0.46	1.12 ± 0.45	9.200	<0.05

Abbreviations: BT, bone transport; PDBFT, pedicled double‐barrel fibular transplantation.

## Discussion

The main findings of this study are that the PDBFT technique brings better clinical effects and fewer complications for the treatment of upper tibial osteomyelitis with bone defects, compared with the BT technique.

### Challenges of Tibial Osteomyelitis with Bone Defects for Surgeons

Tibial osteomyelitis with long segmental bone defects is a serious challenge for trauma surgeons^23^, ^24^. Different methods have been used to treat such patients, which greatly reduces the amputation rate and mortality. The main treatment procedure is staged surgeries, including radical debridement, soft tissue and bone reconstruction. According to the bacterial culture results, high‐grade antibiotics should be used in early stage, with sufficient amount. Sensitive antibiotics should be maintained for at least 6 weeks^25^. Early skin and soft tissue reconstruction should be conducted, too. Healthy soft tissues bring blood supplies and antibiotics to the infected site, and enhance the local anti‐infection ability^26^.

### Advantages and Disadvantages of Ilizarov Bone Transport Technique

Bone reconstruction is another key step in the treatment of tibial osteomyelitis. It still remains controversial for which bone reconstruction method to adopt. Bone transport technique has its unique advantages and is widely used in the treatment of long segmental tibial defects. The repairing range is long and is even close to the full length of the lower leg. It still can be used even if the soft tissue condition is poor. However, there are many postoperative complications for bone transport, including pin‐tract infection, docking site nonunion, axial deviation, and so forth^27^. In addition, the injured limb needs to be fixed for a long time by the annular frame^27^. During the long treatment period, some patients feel extremely uncomfortable, and encounter excessive mental and psychological pressures, resulting in obvious anxiety^28^, ^29^. Several patients even ask for amputation, rather than continuing to wear an Ilizarov annular fixator. Patients' tolerance and compliance limit its application to a certain extent.

### Advantages and Disadvantages of Pedicled Fibular Transplantation

In recent years, vascularized bone transplantation has once again attracted surgeons' attention and interest. The vascularized bone blocks can not only be used in bone defect reconstruction, but also enhance the anti‐infection ability of the recipient site. Compared with other bone graft sources, such as the iliac and ribs, vascularized fibular transplantation is more ideal for the treatment of long segmental bone defects^30^. Fibula is a long tubular bone with triangular section. It has good bending resistance and supporting characteristics. After fibular transplantation, the biomechanical environment changes. Under the stimulation of stress, it can continuously form new callus, thicken, and gradually ossify the tibia. Finally, it can partly or fully replace the original tibia. In addition, the fibula is long enough, which is suitable for repairing long bone defects. Some scholars have reported that the fibula can be transplanted in a length of 26 cm successfully^31^. Free fibular transplantation is complicated due to anastomosis. Unlike free transplantation, it is safe and reliable for ipsilateral pedicled fibular transplantation. However, single‐barrel fibular transplantation has limited supporting strength. Although the fibula can gradually thicken under stress stimulation, the fatigue fracture incidence is high^32^, ^33^.

### Advantages of the PDBFT Technique

The authors used PDBFT technique in the treatment of upper tibial osteomyelitis with bone defects. A locking compression plate was used to bridge defects. Based on our results and experience, good clinical effects were achieved using the PDBFT technique. The supporting strength of double‐barrel fibula is better than that of single fibula, and the fatigue fracture incidence is significantly reduced^33^, ^34^. The authors' retrospective study showed that, compared with BT group, patients in PDBFT group had shorter cure time, better limb functions, better postoperative mental and psychological status, and lower complication incidence. Several scholars used double‐barrel free fibular transplantation to treat patients with femoral and tibial defects, and achieved good results^35^. The first comparison between fibular transplantation and Ilizarov technique for the treatment of tibial osteomyelitis with bone defects was carried out by Yokoyama *et al*. in 2001^36^. They included only eight cases. As the cases were very limited in their study no clear differences between the two techniques could be determined. El‐Gammal *et al*. also compared free fibula graft technique with Ilizarov technique^37^. Based on their experience, when the defect length was <12 cm, Ilizarov bone transport was recommended, and when it was >12 cm, free fibula graft technique was the first option. In this study we reported the comparative results of PDBFT and BT for the treatment of upper tibial osteomyelitis with bone defects. The stability of the defect site is good when the PDBFT technique is used with the aid of a plate. Patients can start knee and ankle joint exercise immediately after operation. This provides a guarantee to maintain good joint functions and reduces the risk of knee joint stiffness and foot drop. After the defects initially heal, the injured limb could partially bear weight and this shortens the whole treatment cycle. Moreover, compared with an external fixator, patients have higher tolerance for an internal plate, and they feel relaxed after the surgery. So the compliance is good, which is conducive to the rehabilitation. A relatively high complication incidence using Ilizarov technique has been reported in previous studies^29^, ^38^. Aktuglu *et al*. summarized the related studies using traditional Ilizarov bone transport in the last 10 years. According to their calculation the mean complication incidence per patient was 1.22^13^. Based on our data, the mean number of complications per patient using bone transport technique was 1.12 ± 0.45. Yet, it was 0.20 ± 0.46 in PDBFT group and the difference was significant. The PDBFT technique showed an obviously lower complication rate than the BT group.

### Limitation of the Study

There are still some deficiencies in the authors' research. The number of patients was limited in both groups, and the follow‐up time was short. We will increase the number of patients and extend the follow‐up time in further studies. The tibial defect range was less than 12 cm in this study. For patients with tibial defects longer than 12 cm, surgeons need to cut off the fibula of both lower limbs to form a double‐barrel shape. The operation is complicated and traumatic. So, further clinical studies are needed to solve this problem.

### Conclusions

Patients were successfully cured by both the PDBFT and BT techniques. Compared with the BT technique, the PDBFT technique brought better clinical effects and fewer complications which could be the first operative choice for the treatment of upper tibial osteomyelitis with bone defects.

## Authors Contributions

All authors listed meet the authorship criteria according to the latest guidelines of the International Committee of Medical Journal Editors, and that all authors are in agreement with the manuscript.

## Funding Information

This study was financially supported by the National Natural Science Foundation of China (81600700) and the National Natural Science Foundation of Shaanxi Province (2022JQ‐757).

## Conflict of Interest

The authors declare that they have no competing interests.
